# Toward Best Practice Guidelines and Curricula for Virtual Care (Telehealth) in a Cancer Center: Protocol for a Multimethod Study

**DOI:** 10.2196/81768

**Published:** 2026-02-20

**Authors:** Sharon Brownie, Lauren Parkinson-Zarb, Andrew Dimech, Maria Ftanou, Patrick Broman

**Affiliations:** 1 School of Rural Medicine, Charles Sturt University, Leeds Parade, Orange, NSW, 2800, Australia, 61 0419330822; 2 School of Health Sciences, Swinburne University of Technology, Johns StreetMelbourne, VIC, 3122, Australia, 61 0419330822; 3 Centre for Health and Social Practice, Waikato Institute of Technology, Hamilton, New Zealand; 4 Department of Nursing, School of Health Sciences, University of Melbourne, Melbourne, VIC, Australia; 5 Office of the Chief Nurse, Peter MacCallum Cancer Center, Melbourne, VIC, Australia; 6 School of Population and Global Health, University of Melbourne, Melbourne, VIC, Australia; 7 Australian Centre for Student Equity and Success, Curtin University, Perth, WA, Australia

**Keywords:** telehealth, telecare, oncology nursing, clinical guidelines, practice guidelines, virtual medicine, nursing education

## Abstract

**Background:**

Virtual health care, originally as telephone-based telehealth, has been used for more than 45 years; however, the literature shows limited understanding of the competencies required for safe virtual care practice in nursing and other health-related fields. This has led to a widening education-practice gap.

**Objective:**

The aim of this study is to identify (1) clinical guidelines for nurses and other health professionals undertaking routine virtual health (telehealth) assessment, triage, and follow-up care; and (2) curricula for preparing health professionals for virtual care. Subsequently, data will be collected within a major cancer treatment service to codetermine core competencies and curricula for nurses engaged in telehealth clinics.

**Methods:**

This was a phased multimethod study including reviews of existing literature, followed by qualitative (in-depth interviews, n=20) and quantitative (online survey, n=200) data collection and co-design workshops (n=5) to achieve project aims. Implementation will involve a pilot and an evaluation before full rollout of the developed guidelines and syllabus.

**Results:**

Literature reviews completed in the initial phase of this project confirm a paucity of existing guidelines for virtual health assessment and an urgent need to develop telehealth or virtual care competency frameworks and curricula for health professionals in training or practice. We propose an approach to develop and test these materials in practice. A total of US $52.6 was provided by a philanthropic alumni for support over the full duration of the project. Recruitment and collection from human participants will commence on February 1, 2026, following procurement of ethics clearance. Phase 2 data collection and analysis will occur from February to December 2026. Results will be presented to the ethics committee and clearance gained to implement and evaluate the program in 2027.

**Conclusions:**

Working with health providers, consumers, and academics toward standards of practice and curricula is clearly needed to ensure that the current and future nursing workforce is prepared for the continuing rise in virtual care. Completion of this project will fill an existing gap in the provision of guidelines and education for nurses providing virtual care.

## Introduction

### Background

Virtual models of care, including telephone and video-based delivery, have been used for more than 4 decades [1-4]; however, literature describes sluggish uptake in embedding newly emerging telehealth modalities in nursing education and practice [5-7].

Though well-established even before the pandemic [8,9], telehealth service delivery accelerated during COVID-19 in response to the urgent need for innovations in care provision, particularly for those unable to access traditional services under stay-at-home orders or during periods of community lockdown [9,10]. The trend for greater use of virtual modalities is reflected globally in patient expectations, health service delivery, and clinical practice [11] as well as in the Australian and New Zealand context [12-14].

While various nursing-related telehealth guidelines or pilot initiatives were developed during the COVID-19 pandemic [15,16], and some work has progressed to better define telehealth education and practice standards [8,17,18], there is no international or national agreement as yet on the education of current and future nurses in telehealth. It is unclear which telehealth competencies are needed, and telehealth placements are not universally recognized as part of required clinical hours. The World Health Organization is promoting telehealth initiatives with a focus on improving access to quality care, equity, resources, and universal health coverage. Despite this, there remains no global standard clinical guidelines or competency descriptions for safe telehealth practice [19].

In most contexts, a significant disconnect exists between the reality of practice and professional education, with content related to safe practice in virtual care effectively absent from nursing accreditation and regulatory standards, nursing scopes of practice, and undergraduate nursing curricula [7,20], despite various nurse leaders, researchers, and policymakers having recommended provision of clinically focused telehealth-related education in nursing and health professional programs [17,21,22].

### Definitions

Terminology related to virtual care can be confusing. Many terms are linked to the delivery of care outside of traditional face-to-face delivery. It is not uncommon for digital health and telehealth to be used interchangeably, so it is important that the definition is clear. Chen et al [23] propose the term “connected health” as an ecosystem or framework to help with definitions and better understand who and what is connected to whom within the rapidly emerging digital health landscape and multiplicity of terminology.

**Figure 1.**
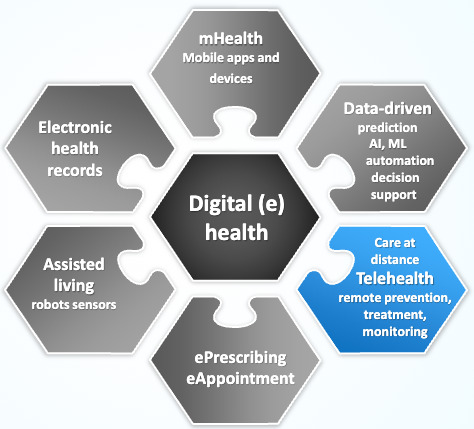
Telehealth within digital (e) health. Adapted from OECD Health Working Paper 129 Socha-Dietrich [25]. AI: artificial intelligence; ML: machine learning; OECD: organization for economic cooperation and development.

For the purposes of this project, the term telehealth is used to refer to care provided at a distance (Figure 1). Telehealth fits within a broader framework of digital eHealth, which includes broad-ranging tools and modalities such as mobile health apps, electronic referrals, wearable devices, electronic prescriptions, patient-reported symptom monitoring, and telehealth modalities electronic records [24,25]. Telehealth is a subset of digital health (Figure 1) and a modality by which virtual care or care at a distance is delivered. Telehealth encounters may be telephone-based or video-based. Nurses providing telehealth services can use a range of information sources, assessment techniques, and communication technologies to provide care virtually, that is, in situations where the nurse and the patient are not physically located in the same place [25,26]. This project focuses on telehealth (patient-related care at distance) rather than the broader concept of digital health or eHealth.

### Study Context

**Figure 2.**
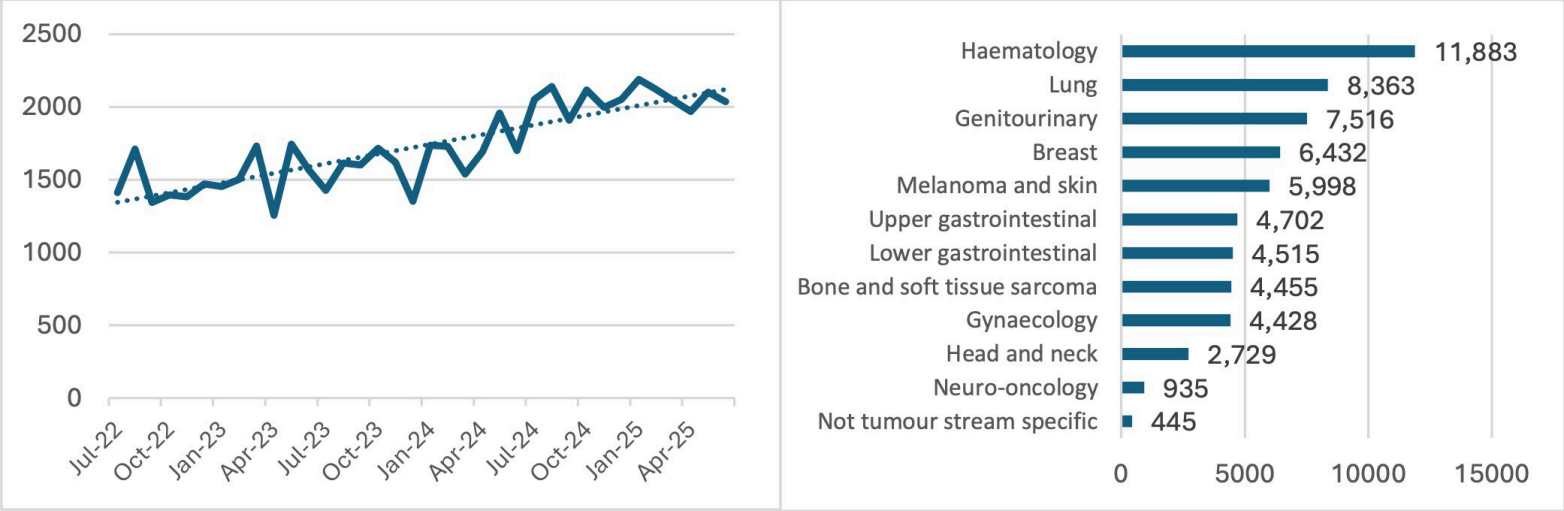
Remote/telehealth consultations at Peter MacCallum Cancer Centre during July 2022 to June 2025. Consultations by month (time series) and total consultation by tumor stream.

The study is performed at the Peter MacCallum Cancer Centre, a large metropolitan cancer treatment center in an Australian city and the leading cancer center in Australia hosting the largest radiation oncology facility in the Southern Hemisphere [27]. It has recently been listed among the top 20 global leaders in cancer care [28]. At Peter MacCallum Cancer Centre, nurse-led telehealth clinics are currently offered across several tumor streams, including gastrointestinal (upper and lower), lung, head and neck, hematology, gynecology, and genitourinary. The total number of nurse-led telehealth consults has grown steadily during 2022‐2025, with 22,627 consultations provided in the 2024 calendar year (Figure 2).

Delivery of telehealth services has increased from 1450 per month in the second half of the year 2022 to 2100 per month in the first half of the year 2025 (Figure 2). While it is the most experienced nurses providing care via this modality, there are currently no formalized guidelines and orientation processes for nurses using telehealth to assess patients and deliver care. Nurses are currently assigned to telehealth services based on their experience and expertise but are not provided with formal orientation or guidelines related to telehealth due to the lack of extant material in the literature or available practice guidelines. This raises several potential quality and safety-related issues, which this project is designed to address.

### Project Rationale and Aims

The project rationale is informed by the increasing use of telehealth services and the realization that the use of these technologies is a core competency within the nursing and broader health workforce [9,11,21]. Telehealth involves the use of information, communication, and assessment techniques to provide health care when clinicians and clients are not physically present in the same location [26]. In a virtual assessment context, the clinician is unable to use all senses: “touch” and “smell” are not available. Patient assessment via digital modalities requires different skill sets than in-person assessments; thus, there is a need for guidelines to ensure clinical safety and quality in telehealth delivery. This study protocol was designed to address current gaps in telehealth delivery and formalize orientation and operating guidelines for nurses assigned across the services.

**Figure 3.**
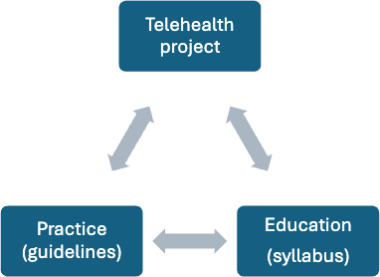
Telehealth project.

The overarching aim is to develop nursing practice guidelines and syllabi for virtual health assessment and care within a major cancer center. The aims are both clinically practice-oriented and education-focused, as shown in Figure 3.

Practice-related aims include the following: (1) identify current practices and practice guidelines in virtual health assessment; (2) identify required nursing competencies for safe practice in telehealth assessment and nursing practice within a cancer center; (3) develop telehealth practice guidelines for senior registered nurses entering telehealth practice in oncology settings, inclusive of triage, health assessment, and routine follow-up; and (4) ensure that the developed guidelines provide the basis for consistent standards and safe practice for telehealth nursing services

Education-related aims include the following: (1) locate existing information about the competencies needed to undertake virtual health assessment and the educational curricula needed to support the development of competencies; (2) detail a syllabus and curriculum-related requirements for the development of the identified oncology telehealth competencies; (3) develop learning objectives, course content, learning outcomes, and assessment tools; (4) design program evaluation guidelines; and (5) pilot and evaluate the developed syllabi and curriculum content.

## Methods

### Study Method

The study method uses a phased multimethod approach, commencing with a review of existing literature followed by qualitative in-depth interviews, online surveys with quantitative methodologies, and co-design workshops. Implementation will involve a fully evaluated pilot before full rollout of the developed guidelines and syllabus. Generative AI was not used in the generation of the protocol or this manuscript.

**Figure 4.**
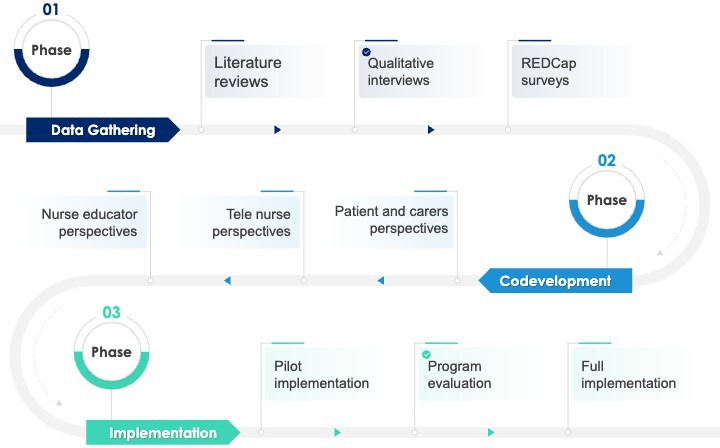
Phased project design. REDCap: Research Electronic Data Capture.

A multimethod approach has been chosen to provide a more comprehensive and robust picture in a context of complexity. Multimethod research uses distinct methods within a single study to strengthen validity by collecting various types of data, which can result in deeper and richer insights and is reported to more effectively address complex issues and enhance applicability of findings [29-31]. The study design involves 3 distinct phases as shown in Figure 4, commencing with data-gathering activities (Phase 1) followed by codevelopment processes (Phase 2). A third phase focuses on program implementation, commencing with pilot testing of the developed virtual health practice guidelines, syllabi, and curricula content. Findings from each phase inform the ongoing development of the project.

The sequential/stepped gathering of data is the inputs for the development of the telehealth practice guidelines, teaching syllabi, and curriculum content for piloting in practice.

### Quality Guidelines

Quality guidelines were integrated throughout the research process to ensure rigor and high standards in the research endeavor, while also enhancing transparency and reproducibility. Subsequently, each phase of the project is designed in accordance with relevant guidelines for quality reporting and conduct of research, including the Joanna Briggs Institute (JBI) guidelines for systematic review and research syntheses [32], PRISMA-ScR (Preferred Reporting Items for Systematic Reviews and Meta-Analyses Extension for Scoping Reviews): checklist and explanation [33], PRISMA (Preferred Reporting Items for Systematic Reviews and Meta-Analyses) reporting guidelines [34,35], JBI criteria for qualitative research [36], and the CASP (Critical Appraisal Skills Program) checklist for quality cross-sectional studies as published by the UK-based CASP [37].

Q-Sort methodology will be used to provide structure, transparency, and credibility to the co-design workshops [38-41]. The program logic model (PLM) will be adopted in the framework for the overall implementation and evaluation of the initiative [42,43], with development and evaluation of the educational products supported by the Kirkpatrick conceptual model that provides a useful tool to measure learner reaction, level of learning, behavior, and results [44].

### Ethical Considerations

### Study Approval

The literature reviews were scheduled to provide insights to inform study design and to protect against duplication of existing knowledge. Reviews were able to proceed without formal ethics approval, following which a formal project design was confirmed, and ethical approval was sought and secured as follows. Project No.: 116964, Ethics No.: 25/60L, HREC Reference: HREC/116964/PMCC.

#### Informed Consent

To ensure that participants do not feel obligated to participate, the study team will inform invitees that participation is voluntary and that they may stop their involvement in the project at any time. The team will explain that their decision will not impact their care or relationships with hospital staff.

#### Data Storage and Privacy Issues

Data will be securely stored and only the research team can access it per the National Statement on Ethical Conduct in Human Research [45] and the Australian Code for Responsible Conduct of Research [46]. Participants’ identities will stay anonymous, and all data will be destroyed 5 years after publication.

No harm is expected. If a participant is at risk, safety procedures will be followed, and a trusted person or clinician may be informed.

#### Consumer Compensation

As per the policy guidelines of the health entity, all participating consumer and community representatives will be compensated for their time. For this purpose, $10,000 AUD (US $7007.8) has been ring-fenced in the budget.

#### Distressed Participants

Distress is not expected during the research. However, if participants become upset, trained project team members will contact them to offer support. Support options may include Cancer Council Victoria counseling or the Peter Mac Employee Assistance Program.

### Phase 1: Data Gathering

#### Literature Reviews

As previously described, the initial phase of the research involved undertaking 2 literature reviews: (1) a practice-oriented scoping review to identify current practices and practice guidelines in virtual health assessment [47]; (2) an education-focused scoping review to locate existing information about the competencies needed to undertake virtual health assessment and the educational curricula needed to support the development of competencies (Brownie et al, unpublished data, 2025).

The literature searches informing this study adhered to JBI guidelines for systematic review and research syntheses [32] and the PRISMA-ScR: checklist and explanation [33]. Search results were reported in accordance with the PRISMA framework reporting guidelines [34,35]. The rigor and quality of the work were subsequently assessed and confirmed through peer review and publication in Q1-ranked journals (Brownie et al, unpublished data, 2025) [47].

#### Qualitative Interviews

In this second phase, 20 participants with lived or professional experience—such as consumers, carers, oncology nurse consultants, educators, academics, and instructional designers—will be interviewed about their experience with telehealth. Insights from these interviews will help shape questions for later online surveys.

During the interviews, participants will be asked about their telehealth experiences, the competencies required for telehealth delivery, ways to improve it, and recommendations for better design, teaching, and service delivery. Each interview will last up to 50 minutes and will be audio-recorded and transcribed (Textbox 1).

Textbox 1. **Participant inclusion and exclusion criteria: qualitative interviews.**

Inclusion criteria:

- Persons with cancer who have completed treatment more than 3 months ago and who have engaged with Peter Mac telehealth services
- Carers of people with cancer
- Telenursing staff and nurse educators working in oncology, with at least 3 years of nursing experience
- Able to engage with researchers in English
- Aged 18 years or older

Exclusion criteria:

- Aged less than 18 years
- Nurses with less than 3 years’ experience
- Nurses with less than 3 years of nursing experience
- Non-English speakers

Qualitative interviews will follow the JBI criteria for qualitative research to ensure rigor and quality in this phase of the research process. The use of JBI guidelines helps ensure robust, credible, and practical approaches to qualitative data collection, while supporting a structured, transparent, and rigorous approach to evidence synthesis [36].

Participants for qualitative interviews will be recruited in 2 ways: (1) through health service consumer and carer groups or newsletters; (2) through health service staff who identify potential participants, such as patients who have completed treatment or carers, including those who traveled from regional areas.

Interested participants will receive an email invitation and can respond by phone or email.

All nursing staff involved in outpatient telehealth clinics will be invited to join the interview via email from the chief investigator. Nursing educators and academic faculty will be invited through the Nursing Department Chair.

After giving consent, participants will be contacted to schedule a time for the interview and will receive a REDCap (Research Electronic Data Capture) survey link to provide basic demographic information. This survey, taking about 5 minutes, is only for describing the participant group (eg, in publications). Textbox 2 lists the information that will be collected.

An experienced qualitative researcher will facilitate the interviews. Interviews will be audio-recorded and transcribed for later reference. Only project managers will have access to recordings, in accordance with the National Statement on Ethical Conduct in Human Research 2023 and the Australian Code for Responsible Conduct of Research 2018 [46].

A coding guide will be developed, and transcripts will be coded into patterns, themes, and key insights. Interviews will be coded by 2 or more coders to ensure consistency of coding and interrater reliability.

Textbox 2. **Demographic data.**

Consumers:

- Gender
- Age
- Country of birth
- Postcode
- Diagnosis
- Date of diagnosis
- Treatments received

Nursing staff, academic faculty, and/or instructional designer:

- Gender
- Age
- Ethnicity
- Years of practice
- Practice area/specialty

#### Patient REDCap Survey

For the third study phase, the project team aims to survey 200 consumers to explore their experiences with receiving telehealth services with survey questions designed to gain further insight into the themes identified by the preceding qualitative interviews.

The survey instrument will be designed with reference to the CASP checklist for quality cross-sectional studies as published by the UK-based CASP [37]. Use of the checklist is an aid to develop and systematically assess the trustworthiness, quality, and relevance of the survey instrument. The checklist provides a structured framework to identify and avoid methodological limitations and potential bias [48].

The target sample size of 200 respondents was determined to ensure sufficient statistical power to detect meaningful effects in user perceptions of telehealth services. Power calculations were based on conventional thresholds for social and behavioral research [49], assuming a medium effect size (Cohen *d*=0.5), a 2-tailed α level of .05, and statistical power (1–β) of 0.80. Under these parameters, a minimum of 128 participants is required to detect significant group differences or associations in primary analyses. To account for potential nonresponse, incomplete data, and subgroup analyses, the sample was increased by approximately 50%, yielding a target of 200 completed responses. This sample size affords adequate precision for estimating proportions within a ±7% margin of error at the 95% confidence level (n=196), which is acceptable for consumer research in health services contexts [29].

No comprehensive or validated instruments suitable for use in the survey were located during extensive searches of the literature (Brownie et al, unpublished data, 2025) [47]. Instead, survey items for this domain will be developed by the research team in consultation with telehealth practitioners at the study site. These items relate to existing frameworks and prior literature and aim to capture relevant dimensions of conceptual validity with telehealth practice. To maximize face validity, the survey will undergo pretesting with a small group of users (n=5) to assess clarity, relevance, and comprehensibility of items. Feedback will be used to refine wording, response options, and survey flow.

As per the agreed ethics clearance, the draft survey will be forwarded to the responsible committee for review. The survey will be designed to take no more than 20 minutes to complete, and time will be factored into the survey design so as not to burden consumers.

To maximize face validity, the survey will undergo pretesting with a small group of users (n=5) to assess clarity, relevance, and comprehensibility of items. Feedback will be used to refine wording, response options, and survey flow (Textbox 3).

Over a 15-week period, patients in various telehealth clinics will be asked if they want to take part in a survey about their experience. Interested participants will get a consent form and survey link through REDCap. Participation is voluntary, and participants will be paid according to health service guidelines. Survey results will be analyzed to help plan the next phase of the project.

Textbox 3. **Participant inclusion profile Research Electronic Data Capture survey.**

Survey participant inclusion profile:

- Persons with cancer who have completed treatment more than 3 months ago
- Persons who have received telehealth sessions from a nurse
- Able to engage with researchers in English
- Aged 18 years or older

#### Codevelopment Workshops

**Figure 5.**
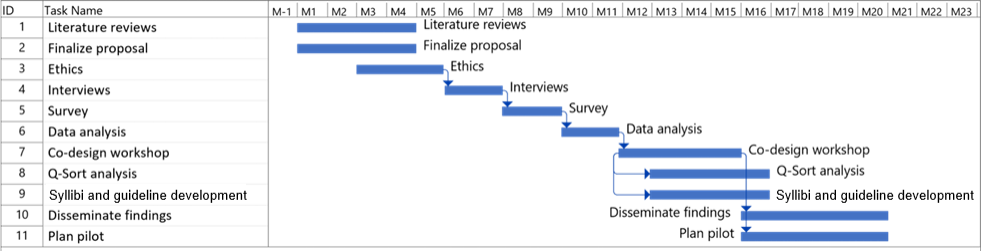
Key project activities.

Following the survey, a series of co-design workshops (Figure 5) will be undertaken, adopting a codevelopment process based on Q-Sort methodology [38,41,50]. These workshops aim to reach agreement on telehealth guidelines, clinical competency requirements, and curricula. Consensus will be built through a series of workshops, using information from Phase 1, including the literature reviews (Brownie et al, unpublished data, 2025) [47], qualitative interviews, and survey findings to inform the development of statements that participants will sort during workshop discussions.

Q-Methodology (Q-Sort) supports systematic information gathering to analyze potentially diverse participant viewpoints in each workshop. Q-Sort is helpful in understanding the perspectives of participants who are likely to have different viewpoints about a topic [38,41]. Q-Sort methodology has been chosen because it will help identify the diversity and commonality of perspectives held by educators, Aboriginal and Torres Strait Islander people, nurses, other health professionals, patients, and families. Q-Sort increases the transparency and replicability of the research. Five basic steps are involved in the Q-Sort methodology [38,41]:

1. Define the domain of focus for discussion
2. Develop a set of statements that participants will sort
3. Select participants representing different groups
4. Q-Sort by participants
5. Analysis and interpretation of information from participants

The workshops will allow participants to view deidentified perspectives which have emerged from Phase 1 findings. Two project team members will facilitate each in-person workshop, which will last about 2 hours (total 10 h). Workshops will be partly guided by participants and will include the following components:

- Presenting the project objectives
- Explanation of the Q-Sort methodology
- Sort and prioritize statements related to the following:
  - Competency requirements for virtual health assessment
  - Requirements for safe practice
  - Curricula content for teaching virtual health assessment
  - Competencies development and upskilling methods
  - Assessment process
  - Delivery and format of curricula
- Opportunity to review materials as they are developed between workshops
- Opportunity for participants to contribute any additional relevant opinions or information

Workshops will be held in person, with options to join by teleconference or through a one-on-one phone discussion if required. Eligibility for participation follows the same criteria as the earlier qualitative interviews (Textbox 1). We plan to recruit 10 consumers (patients and carers) and 10 professionals (nurses, educators, and instructional designers).

Beliefs, views, assumptions, and matters of social or personal importance are inherently subjective [40]. It is anticipated that a range of differing perspectives and viewpoints will exist within Phase 1 findings with potential divergency of views at the workshops. Q-Sort has been chosen as it allows for subjectivity and emphasizes consensus. The approach follows a well-defined and formally structured process [39] without the need for participants to engage in written or spoken activities. Rather, participants rank written statements, objects, pictures, or video clips. Quieter, vulnerable, and potentially marginalized participants can all participate equally in an ethical, nonthreatening, and respectful manner [38,40,51].

Findings from the earlier phases will be consolidated into formal syllabi and practice guidelines, and additional ethics approval will be sought to proceed with the pilot phase. While Q-Sort will provide clear guidance on the priorities for inclusion in both the practice guidelines and curriculum, a conceptual model is needed to take these through to program development. The Kirkpatrick model will be used to analyze and synthesize sorted priorities, agree with the program objectives and course materials, confirm content relevance, and identify the knowledge required of the educational facilitators who will deliver the program [44].

#### Phase 1 and Phase 2 Project Step Timelines

Key activities involved in the first two phases of the project will be undertaken across a 20-month period as shown in Figure 5.

#### Implementation and Evaluation

The final phase of the project involves implementation activities, including the program pilot, evaluation, and subsequent scale-up and full rollout of the developed resources. The pilot will test these materials on a limited scale within the partner university and cancer center.

Evaluation of the overall project phase will be assessed within the overarching PLM—a framework recommended by the Victorian Department of Health that oversees health service delivery at the study setting [43]. The PLM supports the development of a roadmap of inputs, activities, outputs, and outcomes that will guide the entire evaluation process. As per their guidelines for evaluation [42], the study will evaluate project outcomes from the perspective of those impacted by the intervention, specifically consumers, nurses, and educators.

Project outcomes link directly to the research aim. Specifically, project outcomes will result in the development of nursing practice guidelines and syllabi for virtual health assessment and care within a major cancer center. Outcomes will be both clinically oriented and education-focused, as shown in Figure 3.

Evaluation of the developed education and practice products will be supported by the Kirkpatrick conceptual model which provides a useful tool to measure learner reaction, learning, behavior, and results [44]. Feedback from this evaluation will inform subsequent revision of the developed products.

## Results

The literature review conducted in the first stage of the study (Brownie et al, unpublished data, 2025) [47] revealed a lack of comprehensive guidelines for virtual health assessment and competency development, highlighting an urgent need to design telehealth curricula that enhance clinical competency.

These early findings support the project rationale and confirm the relevance of its aims. The reviews also provide assurance that the project will extend rather than duplicate existing knowledge. Findings from the reviews have undergone independent peer review and open-access publication, adding rigor to the research process, strengthening the project rationale, and establishing the foundation for the overall project design.

Data from interviews and the consumer survey will guide discussions in co-design workshops. The workshops will produce consensus statements that prioritize content for telehealth guidelines and curricula. These statements will help define telehealth competencies and develop guidelines and curricula, which will be piloted and evaluated before broader use.

A total of US $52.6 was provided by a philanthropic alumni for support over the full duration of the project. Recruitment and collection from human participants commences February 1, 2026, following procurement of ethics clearance. Phase 2 data collection and analysis will occur from February to December 2026. Results will be presented to the ethics committee and clearance gained to implement and evaluate the program in 2027.

## Discussion

### Anticipated Findings

In 2019, the Australian Federal Government Department of Health and Aged Care commissioned an independent report focused on the educational requirement for the future nursing workforce [52]. This “Educating the Nurse of the Future” report emphasized the way information technology, digital innovation, and telehealth modalities have permeated every aspect of health care development and delivery [52]. It recommended that it would be important to include specific advice within revised registered nurse accreditation standards so that health education providers would be clear about what aspects of digital health technologies and informatics should be included in curricula and the specific skill level and competencies nurses should be expected to achieve [52].

Since the COVID-19 pandemic [8,9], telehealth service delivery has only accelerated in response to the urgent need for innovations in care provision, particularly those unable to access traditional service while under stay-at-home orders and during periods of enforced community lockdown [9,10]. The trend for greater use of digital modalities is reflected globally in patient expectations, health service delivery, and clinical practice [11] across Australia and New Zealand [12-14].

Despite this, many nurses in telehealth practice have had no formal training [53]; nursing academics are reported as having failed in the job of preparing nursing graduates for roles in telehealth [21]; experience and content across the various levels of nursing education programs appears low [5]; and graduates are entering the health workforce without telehealth education and the required virtual practice competencies [20]. An Australian national study funded by the Victorian Higher Education State Investment Fund reported 69.9% of undergraduate nursing (entry to practice) programs as having no digital health content. Of further concern, findings report a significant gap in the confidence and knowledge of nurse academics to teach digital- and telehealth-related content [54].

Regulatory gaps are also evident. While Australia’s recently revised Registered Nurse accreditation standards [55] record feedback detailing industry expectations regarding the inclusion of a “focus on digital health.” Subsequently, the updated standards support inclusion and embedding of 2 subjects within curricula—health informatics and digital health—and that the National Informatics Standards [56] be accessed as the source from which health education providers should elicit competencies and learning outcomes [55]. Developed by the Australian Nursing and Midwifery Federation (2015), the National Information Standards for Nurses and Midwives focus on 3 key domains: computer literacy, information literacy, and information management [56]. Specific clinical process and competency requirements related to telehealth-based, “virtual clinical health assessment” and service delivery are not included.

Other existing guidelines similarly focus primarily on the technological aspects of health service delivery such as computer literacy, information literacy, and information management [56] and/or digital professionalism, leadership and advocacy, data and information quality, technology, and information-enabled care [13]. The Australian Health Practitioners Regulatory Authority has published telehealth guidance for practitioners, primarily providing a definition of telehealth services and who can use telehealth [57]. In contrast, this project is clinically focused and aims to fill current gaps related to the actual requirements for safe nursing practice in virtual health.

Undoubtedly, various Australia-based schools of nursing will be updating curricula in response to the revised 2019 accreditation standards, but in the absence of standardized Australian Health Practitioners Regulatory Authority or the Australian Nursing and Midwifery Accreditation Council guidelines, developments are likely to be variable. The importance of the issue is emphasized in the Australian Government’s 2021 response to the Educating the Nurse of the Future report [12]. Recommendation 20 of 26 states:

*ANMAC’s RN accreditation standards for health informatics and digital health technologies should specify learning outcomes and the level of expertise required. The EN accreditation standards should contain similar specifications.* [12]

[p 16]

However, many nurses working in telehealth have received little to no formal training [53]. Nursing educators have been criticized for not adequately preparing graduates for telehealth roles [21], and the amount of telehealth content and practical experience included across nursing education programs remains limited [5]. Subsequently, most graduates are entering the workforce without the essential competencies needed for telehealth practice [20].

The development imperative is compelling. This project is designed to work with clinicians, educators, and consumers in the development of telehealth clinical practice guidelines and curricula to address the gap in virtual care delivery.

### Strengths and Limitations

This single-site study limits generalizability, and the authors acknowledge potential bias in co-design workshops wherein some participants may be more vocal than others and/or the workshop participant group may be unintentionally skewed in comparison to the overall population focus of the study. However, the mixed methods approach, with active input from consumers and clinicians, and the use of Q-Sort methods will serve to minimize these limitations.

### Conclusions

Working with health providers, consumers, and academics in modernizing standards of practice and curricula and upskilling nurse educators is clearly needed to ensure that the current and future nursing workforce is prepared for the continuing rise in telehealth services. Completion of this industry/education co-design project will fill the existing gap in the provision of clinically oriented guidelines and education for nurses providing telehealth services with care at distance.

## Abbreviations

JBI: Joanna Briggs Institute
PLM: program logic model
PRISMA: Preferred Reporting Items for Systematic Reviews and Meta-Analyses
PRISMA-ScR: Preferred Reporting Items for Systematic Reviews and Meta-Analyses extension for Scoping Reviews
REDCap: Research Electronic Data Capture
